# Author Correction: Chromatin compartmentalization regulates the response to DNA damage

**DOI:** 10.1038/s41586-023-06841-8

**Published:** 2023-11-16

**Authors:** Coline Arnould, Vincent Rocher, Florian Saur, Aldo S. Bader, Fernando Muzzopappa, Sarah Collins, Emma Lesage, Benjamin Le Bozec, Nadine Puget, Thomas Clouaire, Thomas Mangeat, Raphael Mourad, Nadav Ahituv, Daan Noordermeer, Fabian Erdel, Martin Bushell, Aline Marnef, Gaëlle Legube

**Affiliations:** 1 MCD, Centre de Biologie Intégrative (CBI), CNRS, Université de Toulouse, UT3, Toulouse, France; 2 Department of Bioengineering and Therapeutic Sciences, University of California, San Francisco, San Francisco, CA USA; 3 Institute for Human Genetics, University of California, San Francisco, San Francisco, CA USA; 4 Cancer Research UK Beatson Institute, Glasgow, UK; 5 LITC Core Facility, Centre de Biologie Integrative, Université de Toulouse, CNRS, UPS, Toulouse, France; 6 Université Paris-Saclay, CEA, CNRS, Institute for Integrative Biology of the Cell (I2BC), Gif-sur-Yvette, France; 7 Institute of Cancer Sciences, University of Glasgow, Glasgow, UK

**Keywords:** Nuclear organization, DNA damage and repair

Correction to: *Nature* 10.1038/s41586-023-06635-y Published online 18 October 2023

In the version of the article initially published, the values on the colour bars in Fig. 1a were inverted. This has been corrected in the HTML and PDF versions of the article.

