# Supplementary material for: Chromatin compartmentalization regulates the response to DNA damage
Source: Nature. 2023 Oct 18;623(7985):183–92. doi: 10.1038/s41586-023-06635-y (PMC10620078; doi:10.1038/s41586-023-06635-y)
Supplement: Supplementary file 1 — Uncropped gels for Extended Data Figs. 7c and 8a,b. [file 41586_2023_6635_MOESM1_ESM.pdf]

nature portfolio

https://doi.org/10.1038/s41586-023-06635-y

# Supplementary information

# Chromatin compartmentalization regulates the response to DNA damage

In the format provided by the authors and unedited

## SI guide

# Chromatin compartmentalization regulates the response to DNA damage

Coline Arnould[1#], Vincent Rocher[1#], Florian Saur[1], Aldo S. Bader[2], Fernando Muzzopappa[1], Sarah Collins[1], Emma Lesage[1], Benjamin Le Bozec[1], Nadine Puget[1], Thomas Clouaire[1], Thomas Mangeat[3], Raphael Mourad[1], Nadav Ahituv[4,5], Daan Noordermeer[6], Fabian Erdel[1], Martin Bushell[2,7], Aline Marnef[1], and Gaëlle Legube[1]*

1. *MCD, Centre de Biologie Intégrative (CBI), CNRS, Université de Toulouse, UT3*
2. *Cancer Research UK Beatson Institute, Garscube Estate, Switchback Road, Bearsden, Glasgow G61 1BD, UK.*
3. *LITC Core Facility, Centre de Biologie Integrative, Université de Toulouse, CNRS, UPS, 31062 Toulouse, France*
4. *Department of Bioengineering and Therapeutic Sciences, University of California, San Francisco, San Francisco, CA, 94158, USA*
5. *Institute for Human Genetics, University of California, San Francisco, San Francisco, CA, 94158, USA*
6. *Université Paris-Saclay, CEA, CNRS, Institute for Integrative Biology of the Cell (I2BC), 91198, Gif-sur-Yvette, France*
7. *Institute of Cancer Sciences, University of Glasgow, Garscube Estate, Switchback Road, Bearsden, Glasgow G61 1QH, UK*

## Legends of Supplementary Videos 1-6

## Legend of Supplementary Figure 1

**Supplementary Video 1: Live-imaging using random illumination microscopy (RIM) in 53BP1-GFP DIvA cells. Example 1**

3D Time-series super-resolved movie by RIM microscopy around a small nuclear volume (8µm*6µm*2µm) of 53BP1-GFP reconstructed at 40-s intervals during 34min.

**Supplementary Video 2: Live-imaging using random illumination microscopy (RIM) in 53BP1-GFP DIvA cells. Example 2**

3D Time-series super-resolved movie by RIM microscopy around a small nuclear volume (5µm*5µm*2µm) of 53BP1-GFP reconstructed at 40-s intervals during 21min and 20s.

**Supplementary Video 3: Live-imaging using random illumination microscopy (RIM) in 53BP1-GFP DIvA cells. Example 3**

3D Time-series super-resolved movie by RIM microscopy around a small nuclear volume (4µm*4.5µm*2.5µm) of 53BP1-GFP reconstructed at 40-s intervals during 32min.

**Supplementary Video 4: Live-imaging using random illumination microscopy (RIM) in 53BP1-GFP DIvA cells. Example 4**

3D Time-series super-resolved movie by RIM microscopy around a small nuclear volume (5µm*5µm*3.5µm) of 53BP1-GFP reconstructed at 40-s intervals during 40min.

**Supplementary Video 5: Live-imaging using random illumination microscopy (RIM) in 53BP1-GFP DIvA cells. Example 5**

3D Time-series super-resolved movie by RIM microscopy around a small nuclear volume (6µm*5µm*3.5µm) of 53BP1-GFP reconstructed at 40-s intervals during 40min.

**Supplementary Video 6: Live-imaging using random illumination microscopy (RIM) in 53BP1-GFP DIvA cells. Example 6**

3D Time-series super-resolved movie by RIM microscopy around a small nuclear volume (4*2.5*3.5µm) of 53BP1-GFP reconstructed at 40-s intervals during 38min and 40s.

**Supplementary Figure 1: uncropped gels for Extended Data Fig. 7c, Extended Data 8a-b**

# Extended data Fig. 7c

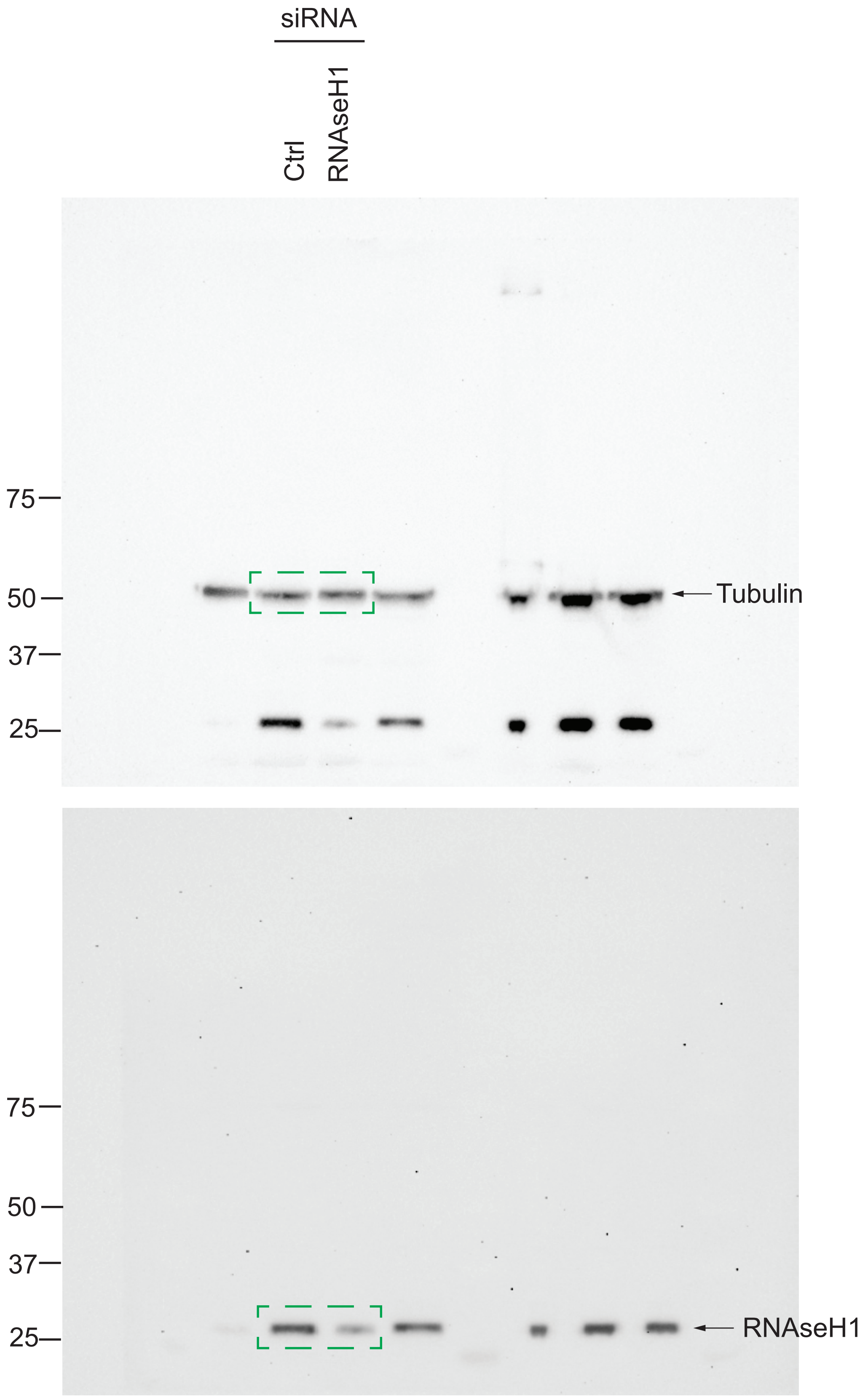


**Supplementary Figure 1. Uncropped Western blots images with size marker indications (page 1)**
Validation of the efficiency of the siRNA against RNAseH1 by Western Blot. Green boxes indicate cropped versions found in the Extended data Figure 7c.

Extended data Fig. 8a

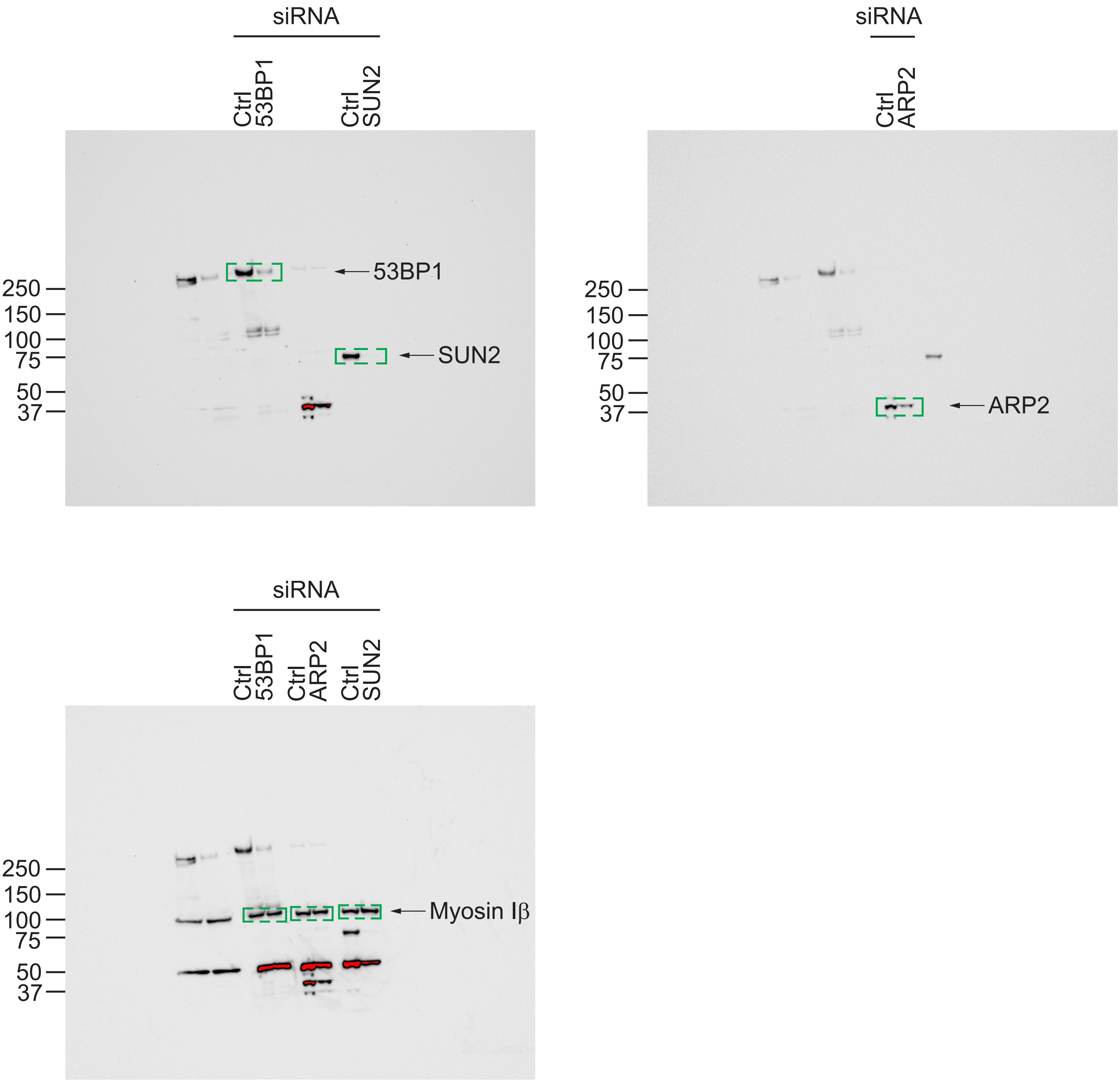


**Supplementary Figure 1. Uncropped Western blots images with size marker indications (page 2)**
Validation of the efficiency of the siRNA against 53BP1 or SUN2 (top left panel) or ARP2 (top right panel) by Western Blot. Green boxes indicate cropped versions found in the Extended data Figure 8a.

# Extended data Fig. 8b

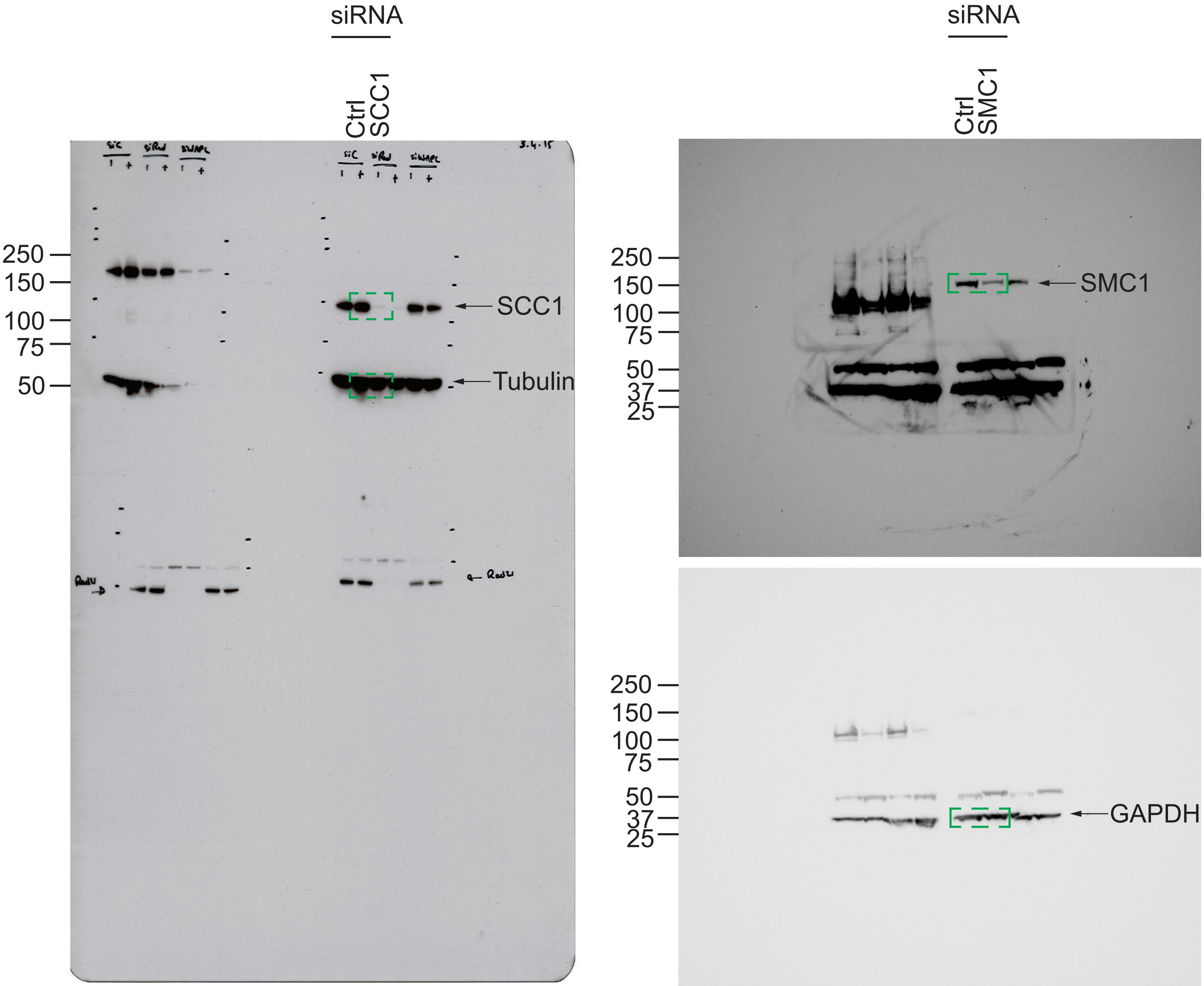


**Supplementary Figure 1. Uncropped Western blots images with size marker indications (page 3)**
Validation of the efficiency of the siRNA against SCC1 (left panel) or SMC1 (right panel) by Western Blot. Green boxes indicate cropped versions found in the Extended data Figure 8b.
