# Supplementary material for: Chromatin compartmentalization regulates the response to DNA damage
Source: Nature. 2023 Oct 18;623(7985):183–92. doi: 10.1038/s41586-023-06635-y (PMC10620078; doi:10.1038/s41586-023-06635-y)
Supplement: Supplementary file 2 — Reporting Summary [file 41586_2023_6635_MOESM2_ESM.pdf]

Corresponding author(s): Gaelle Legube
Last updated by author(s): Aug 3, 2023

Reporting Summary

Nature Portfolio wishes to improve the reproducibility of the work that we publish. This form provides structure for consistency and transparency in reporting. For further information on Nature Portfolio policies, see our Editorial Policies and the Editorial Policy Checklist.

Statistics

For all statistical analyses, confirm that the following items are present in the figure legend, table legend, main text, or Methods section.

n/a	Confirmed	
☐	☒	The exact sample size (*n*) for each experimental group/condition, given as a discrete number and unit of measurement
☐	☒	A statement on whether measurements were taken from distinct samples or whether the same sample was measured repeatedly
☐	☒	The statistical test(s) used AND whether they are one- or two-sided<br>*Only common tests should be described solely by name; describe more complex techniques in the Methods section.*
☒	☐	A description of all covariates tested
☒	☐	A description of any assumptions or corrections, such as tests of normality and adjustment for multiple comparisons
☐	☒	A full description of the statistical parameters including central tendency (e.g. means) or other basic estimates (e.g. regression coefficient) AND variation (e.g. standard deviation) or associated estimates of uncertainty (e.g. confidence intervals)
☐	☒	For null hypothesis testing, the test statistic (e.g. *F*, *t*, *r*) with confidence intervals, effect sizes, degrees of freedom and *P* value noted<br>*Give P values as exact values whenever suitable.*
☒	☐	For Bayesian analysis, information on the choice of priors and Markov chain Monte Carlo settings
☒	☐	For hierarchical and complex designs, identification of the appropriate level for tests and full reporting of outcomes
☐	☒	Estimates of effect sizes (e.g. Cohen's *d*, Pearson's *r*), indicating how they were calculated

Our web collection on statistics for biologists contains articles on many of the points above.

Software and code

Policy information about availability of computer code

Data collection

Bio-Rad CFX Manager version 3.1
ChemiDoc™ Touch Imaging System.
Image Lab Touch version 1.2.0.12
MetaMorph version 7.1.0.0
Micro-Manager 2.0
Colombus software version 2.8.2
Harmony software version 4.9

Data analysis

Bio-Rad CFX Manager version 3.1
Article Github (https://github.com/LegubeDNAREPAIR/ATMcompD)
A custom R script (https://github.com/bbcf/bbcfutils/blob/master/R/smoothData.R) was used to build the coverage file in bedGraph format
Juicer Tools HiCCUPS program (https://github.com/aidenlab/juicer/wiki/HiCCUPS)
Juicer Tools APA program (https://github.com/aidenlab/juicer/wiki/APA)
mProfile (https://github.com/aldob/mProfile)
A specific python script from FourCSeq R package (Klein, F. A. et al. FourCSeq: analysis of 4C sequencing data. Bioinformatics 31, 3085–3091 (2015)) v1.2.0
edgeR package 3.26.0
Image Lab Touch software 5.0
ImageJ (Fiji) v1.53c
Icy 2.4.1.0
Inscoper
HiTC 1.30.0
JASPAR v2020
motifmatchR 1.6.0

igraph R package 1.2.4.1
regioneR R package 1.15.2
GenomicRanges 1.38
plyranges 1.6.10
tidyverse 1.3.0 (including ggplot2 R package 3.3.3)
patchwork 1.1.1
ggforce 0.3.3
ggside 0.2.0
ggtext 0.1.1
Integrated Genome Browser version 9.1.6
Juicer version 1.6
Juicebox version 1.11.08
macs2 2.1.2
macs3 3.0.0a7
bedtools v2.26.0
bwa 0.7.12-r1039
samtools 1.9
deeptools 3.4.3
R 3.6.3
gkmsvm 0.81.0
CellProfiler 4.2.1
Colombus software version 2.8.2

For manuscripts utilizing custom algorithms or software that are central to the research but not yet described in published literature, software must be made available to editors and reviewers. We strongly encourage code deposition in a community repository (e.g. GitHub). See the Nature Portfolio guidelines for submitting code & software for further information.

Data

Policy information about availability of data

All manuscripts must include a data availability statement. This statement should provide the following information, where applicable:

- Accession codes, unique identifiers, or web links for publicly available datasets
- A description of any restrictions on data availability
- For clinical datasets or third party data, please ensure that the statement adheres to our policy

New high throughput sequencing data have been deposited publicly to Array Express under the accession number: E-MTAB-10865.

Other high-throughput sequencing data used in this study are available under accession numbers: E-MTAB-8851 (Hi-C data before and after DSB induction and upon Ctrl or SCC1 depletion; pATM ChIP-seq), E-MTAB-5817 (BLESS before and after DSB induction; LIG4, 53BP1, gH2AX, FK2 and histone H1 ChIP-seq experiments) and E-MTAB-6318 (DRIP-seq before and after DSB induction; SETX ChIP-seq). Breakpoint positions of inter-chromosomal translocations across 18 different cancer types were retrieved from Zhang et al., Cell Rep, 2018 (doi: 10.1016/j.celrep.2018.06.025).

Field-specific reporting

Please select the one below that is the best fit for your research. If you are not sure, read the appropriate sections before making your selection.

☒ Life sciences ☐ Behavioural & social sciences ☐ Ecological, evolutionary & environmental sciences

For a reference copy of the document with all sections, see nature.com/documents/nr-reporting-summary-flat.pdf

Life sciences study design

All studies must disclose on these points even when the disclosure is negative.

Sample size	No sample size calculation was performed. The number of samples in each experiment was determined based on standards practice in the field (N=3). This is why we did N= or > 3 independent experiments unless stated otherwise, for example some sequencing experiments, which based on our experience require less replicates (doi: 10.1038/s41586-021-03193-z ; 10.1016/j.molcel.2018.08.020 ; 10.1038/s41467-018-02894-w etc.). The number of independent experiments are indicated in the legend of each Figure.
Data exclusions	No data were excluded from analysis.
Replication	RNA-seq before and after DSB induction: 2 replicates. Replication was successful<br>Hi-C before and after DSB induction: 3 replicates. Replication was successful<br>Hi-C after DSB induction upon ATM or DNA-PK inhibition : n=1. Data were confirmed by Immunofluorescence experiments (i.e. DSB clustering)<br>Hi-C after DSB induction in CTRL or SETX depleted cells : n=1. Data were confirmed by Immunofluorescence experiments (i.e. DSB clustering)<br>4C-seq before and after DSB induction in the G1, G2 or S phase of the cell cycle: n=1, 4 DSB viewpoints. The same results were observed from the different DSB viewpoints.<br>Amplicon-seq upon Ctrl, SCC1, SUN2 or Arp2 depletion : 4 replicates. Replication was successful<br>Translocation assays (qPCR) : n=3 or 4 depending on the conditions. Replication was successful<br>RT-qPCR after siRNA treatments /etoposide: n=4. Replication was successful<br>RNAscope: n=3 for CCL2 and PPIB probes, n=6 for GADD45A probe. Replication was successful<br>qDRIP-seq: n=1.

	RNAseq siRNA SETX n=2. Replication was successful<br>High-throughput microscopy n=3. Replication was successful<br>DRIP-seq siSETX n=1<br>Half-FRAP n=1 experiment with n=22 foci analyzed. Replication was successful
Randomization	Randomization is not relevant because we did not use different experimental groups in our study.
Blinding	Blinding was not relevant to our study since we did not have experimental group to compare.

Reporting for specific materials, systems and methods

We require information from authors about some types of materials, experimental systems and methods used in many studies. Here, indicate whether each material, system or method listed is relevant to your study. If you are not sure if a list item applies to your research, read the appropriate section before selecting a response.

Materials & experimental systems

n/a	Involved in the study
☐	☒ Antibodies
☐	☒ Eukaryotic cell lines
☒	☐ Palaeontology and archaeology
☒	☐ Animals and other organisms
☒	☐ Human research participants
☒	☐ Clinical data
☒	☐ Dual use research of concern

Methods

n/a	Involved in the study
☒	☐ ChIP-seq
☒	☐ Flow cytometry
☒	☐ MRI-based neuroimaging

Antibodies

Antibodies used	See methods section of the manuscript for use and dilution of antibodies<br><br>Primary antibodies:<br>SUN2 (Abcam ab124916, clone EPR6557)<br>ARP2 (Abcam ab128934, clone EPR7980)<br>53BP1 (Novus Biologicals NB100-305, polyclonal)<br>SCC1 (Abcam ab992, polyclonal)<br>SMC1 (Abcam ab75819, clone EP2879Y)<br>Myosin (Sigma M3567, polyclonal)<br>GAPDH (Sigma MAB374, clone 6C5)<br>DNA-RNA Hybrid (clone S9.6) (Antibodies Incorporation)<br>RnaseH1 (Invitrogen PA5-30974, polyclonal)<br>gH2AX (Millipore Sigma 05-636, clone JBW301)<br><br>Secondary antibodies:<br>Goat Anti-Mouse IgG-Peroxydase (Sigma A2554, polyclonal)<br>Goat Anti-Rabbit IgG-Peroxydase (Sigma A0545, polyclonal)<br>Goat anti-Mouse IgG (H+L) Alexa Fluor™ 488 (Invitrogen # A-11029, polyclonal)<br>Goat anti-Mouse IgG (H+L) Alexa Fluor™ 594 (Invitrogen # A-11032, polyclonal)<br>Goat anti-Rabbit IgG (H+L) Alexa Fluor™ 488 (Invitrogen # A-11034, polyclonal)<br>Goat anti-Rabbit IgG (H+L) Alexa Fluor™ 594 (Invitrogen # A-11037, polyclonal)
Validation	Primary antibodies:<br>SUN2 (Abcam ab124916) previously validated in human for WB as stated by the manufacturers on their website (https://www.abcam.com/sun2-antibody-epr6557-ab124916.html)<br>ARP2 (Abcam ab128934) previously validated in human for WB as stated by the manufacturers on their website (https://www.abcam.com/arp2-antibody-epr7980-ab128934.html)<br>53BP1 (Novus Biologicals NB100-305) previously validated in human for WB as stated by the manufacturers on their website (https://www.novusbio.com/products/53bp1-antibody_nb100-305)<br>SCC1 (Abcam ab992) previously validated in human for WB as stated by the manufacturers on their website (https://www.abcam.com/rad21-antibody-ab992.html)<br>SMC1 (Abcam ab75819) previously validated in human for WB as stated by the manufacturers on their website (https://www.abcam.com/smc1a-antibody-ep2879y-ab75819.html#lb)<br>Myosin (Sigma M3567) previously validated in human for WB as stated by the manufacturers on their website (https://www.sigmaaldrich.com/catalog/product/sigma/m3567?lang=fr&region=FR)<br>GAPDH (Sigma MAB374) previously validated in human for WB as stated by the manufacturers on their website (https://www.sigmaaldrich.com/US/en/product/mm/mab374?gclid=CjwKCAjwxo6IBhBKEiwAXSYBs2mXMTrpnvmfDppcnfh3YEPY6Rdo9QRlHfhswQNLHvcDDPDRyV_wxxoCiykQAvD_BwE)<br>DNA-RNA Hybrid antibody S9.6 antibodies previously validated in human for DRIP experiment (Crossely et al, 2020, NAR, doi: 10.1093/nar/gkaa500)<br>gH2AX (Millipore Sigma 05-636) previously validated in IF as stated by the manufacturers on their website (https://www.emdmillipore.com/US/en/product/Anti-phospho-Histone-H2A.X-Ser139-Antibody-clone-JBW301,MM_NF-05-636-I?

ReferrerURL=https%3A%2F%2Fwww.google.com%2F)

Secondary antibodies:
Goat Anti-Mouse IgG-Peroxydase (Sigma A2554) previously validated for WB as stated by the manufacturers on their website (https://www.sigmaaldrich.com/US/en/product/sigma/a2554)
Goat Anti-Rabbit IgG-Peroxydase (Sigma A0545) previously validated for WB as stated by the manufacturers on their website (https://www.sigmaaldrich.com/US/en/product/sigma/a0545)
Goat anti-Mouse IgG (H+L) Alexa Fluor™ 488 (Invitrogen # A-11029) previously validated for IF as stated by the manufacturers on their website (https://www.thermofisher.com/antibody/product/Goat-anti-Mouse-IgG-H-L-Highly-Cross-Adsorbed-Secondary-Antibody-Polyclonal/A-11029)
Goat anti-Mouse IgG (H+L) Alexa Fluor™ 594 (Invitrogen # A-11032) previously validated in IF as stated by the manufacturers on their website (https://www.thermofisher.com/antibody/product/Goat-anti-Mouse-IgG-H-L-Highly-Cross-Adsorbed-Secondary-Antibody-Polyclonal/A-11032)
Goat anti-Rabbit IgG (H+L) Alexa Fluor™ 488 (Invitrogen # A-11034) previously validated in IF as stated by the manufacturers on their website (https://www.thermofisher.com/antibody/product/Goat-anti-Rabbit-IgG-H-L-Highly-Cross-Adsorbed-Secondary-Antibody-Polyclonal/A-11034)
Goat anti-Rabbit IgG (H+L) Alexa Fluor™ 594 (Invitrogen # A-11037) previously validated in IF as stated by the manufacturers on their website (https://www.thermofisher.com/antibody/product/Goat-anti-Rabbit-IgG-H-L-Highly-Cross-Adsorbed-Secondary-Antibody-Polyclonal/A-11037)

Eukaryotic cell lines

Policy information about cell lines

Cell line source(s)	Cell lines developped from U2OS cells (ATCC® HTB-96™) in the Gaelle Legube's laboratory (DIvA cell line, 53BP1-GFP-DIvA, and AID-DIvA cell line)
Authentication	Authentication of the U20S cell line was performed by the provider ATCC which uses morphology and Short Tandem Repeat profiling to confirm the identity of human cell lines. DIvA, AID-DIvA and 53BP1-GFP-DIvA cells derived from these U20S cells were not further authenticated.
Mycoplasma contamination	All cell lines (DIvA, 53BP1-GFP-DIvA and AID-DIvA) were regularly tested for absence of mycoplasma contamination by using the MycoAlert Mycoplasma (Lonza). All cell lines used in this study were tested negative for Mycoplasma.
Commonly misidentified lines (See ICLAC register)	No commonly misidentified cell lines were used in the study. U2OS are not registered in ICLAC.
